# Exogenous and endogenous angiotensin‐II decrease renal cortical oxygen tension in conscious rats by limiting renal blood flow

**DOI:** 10.1113/JP270731

**Published:** 2016-08-18

**Authors:** Tonja W. Emans, Ben J. Janssen, Maximilian I. Pinkham, Connie P. C. Ow, Roger G. Evans, Jaap A. Joles, Simon C. Malpas, C. T. Paul Krediet, Maarten P. Koeners

**Affiliations:** ^1^ Nephrology and HypertensionUniversity Medical Centre UtrechtUtrechtThe Netherlands; ^2^ Internal Medicine‐NephrologyAcademic Medical Centre at the University of AmsterdamThe Netherlands; ^3^ Department of Pharmacology and ToxicologyMaastricht UniversityMaastrichtThe Netherlands; ^4^ Department of PhysiologyUniversity of AucklandAucklandNew Zealand; ^5^ Cardiovascular Disease Program, Biosciences Discovery Institute and Department of PhysiologyMonash UniversityMelbourneAustralia; ^6^ Millar IncAucklandNew Zealand; ^7^ School of Physiology, Pharmacology and NeuroscienceUniversity of BristolBristolUK

**Keywords:** angiotensin‐II, cortex, Cyp1a1Ren2 transgenic rat, hypoxia, kidney, oxygenation, phenylephrine, telemetry

## Abstract

**Key points:**

- Our understanding of the mechanisms underlying the role of hypoxia in the initiation and progression of renal disease remains rudimentary.
- We have developed a method that allows wireless measurement of renal tissue oxygen tension in unrestrained rats.
- This method provides stable and continuous measurements of cortical tissue oxygen tension $\left(P_{O_{2}}\right)$ for more than 2 weeks and can reproducibly detect acute changes in cortical oxygenation.
- Exogenous angiotensin‐II reduced renal cortical tissue $P_{O_{2}}$ more than equi‐pressor doses of phenylephrine, probably because it reduced renal oxygen delivery more than did phenylephrine.
- Activation of the endogenous renin–angiotensin system in transgenic Cyp1a1Ren2 rats reduced cortical tissue $P_{O_{2}}$; in this model renal hypoxia precedes the development of structural pathology and can be reversed acutely by an angiotensin‐II receptor type 1 antagonist.
- Angiotensin‐II promotes renal hypoxia, which may in turn contribute to its pathological effects during development of chronic kidney disease.

**Abstract:**

We hypothesised that both exogenous and endogenous angiotensin‐II (AngII) can decrease the partial pressure of oxygen $\left(P_{O_{2}}\right)$ in the renal cortex of unrestrained rats, which might in turn contribute to the progression of chronic kidney disease. Rats were instrumented with telemeters equipped with a carbon paste electrode for continuous measurement of renal cortical tissue $P_{O_{2}}$. The method reproducibly detected acute changes in cortical oxygenation induced by systemic hyperoxia and hypoxia. In conscious rats, renal cortical $P_{O_{2}}$ was dose‐dependently reduced by intravenous AngII. Reductions in $P_{O_{2}}$ were significantly greater than those induced by equi‐pressor doses of phenylephrine. In anaesthetised rats, renal oxygen consumption was not affected, and filtration fraction was increased only in the AngII infused animals. Oxygen delivery decreased by 50% after infusion of AngII and renal blood flow (RBF) fell by 3.3 ml min^−1^. Equi‐pressor infusion of phenylephrine did not significantly reduce RBF or renal oxygen delivery. Activation of the endogenous renin–angiotensin system in Cyp1a1Ren2 transgenic rats reduced cortical tissue $P_{O_{2}}$. This could be reversed within minutes by pharmacological angiotensin‐II receptor type 1 (AT_1_R) blockade. Thus AngII is an important modulator of renal cortical oxygenation via AT_1_ receptors. AngII had a greater influence on cortical oxygenation than did phenylephrine. This phenomenon appears to be attributable to the profound impact of AngII on renal oxygen delivery. We conclude that the ability of AngII to promote renal cortical hypoxia may contribute to its influence on initiation and progression of chronic kidney disease.

## Key points

- Our understanding of the mechanisms underlying the role of hypoxia in the initiation and progression of renal disease remains rudimentary.
- We have developed a method that allows wireless measurement of renal tissue oxygen tension in unrestrained rats.
- This method provides stable and continuous measurements of cortical tissue oxygen tension $\left(P_{O_{2}}\right)$ for more than 2 weeks and can reproducibly detect acute changes in cortical oxygenation.
- Exogenous angiotensin‐II reduced renal cortical tissue $P_{O_{2}}$ more than equi‐pressor doses of phenylephrine, probably because it reduced renal oxygen delivery more than did phenylephrine.
- Activation of the endogenous renin–angiotensin system in transgenic Cyp1a1Ren2 rats reduced cortical tissue $P_{O_{2}}$; in this model renal hypoxia precedes the development of structural pathology and can be reversed acutely by an angiotensin‐II receptor type 1 antagonist.
- Angiotensin‐II promotes renal hypoxia, which may in turn contribute to its pathological effects during development of chronic kidney disease.

**Abbreviations**
AngII: angiotensin‐II
AT_1_R: angiotensin‐II receptor type 1
CKD: chronic kidney disease
CV: coefficient of variation
$D_{O_{2}}$: renal oxygen delivery
GFR: glomerular filtration rate
I3C: indole‐3‐carbinol
PE: phenylephrine
$P_{O_{2}}$: tissue oxygen tension
RAS: renin–angiotensin system
RBF: renal blood flow
$Q_{O_{2}}$: renal oxygen consumption
*T*_Na_: tubular sodium reabsorption

## Introduction

Chronic kidney disease (CKD) is associated with low tissue oxygen tension ($P_{O_{2}}$) within the kidney (i.e. renal hypoxia) (Evans *et al*. 2013). For example, in rats renal tissue hypoxia (i.e. $P_{O_{2}}$ < 10 mmHg) was detected using pimonidazole adduct immunohistochemistry in the remnant kidney model of CKD (Manotham *et al*. 2004). Renal hypoxia has also been observed in the early stages of glomerulonephritis in rats (Matsumoto *et al*. 2004). These and other observations led to the proposition that renal parenchymal hypoxia is not just a consequence of kidney disease, but rather a critical pathological mediator, irrespective of the primary aetiology of the disease (Fine *et al*. 2000; Nangaku, 2006; Tanaka *et al*. 2006; Heyman *et al*. 2008; Palm & Nordquist, 2011).

Activation of the renin–angiotensin system (RAS) is an established modulator of the progression of CKD (Remuzzi *et al*. 2005; Kobori *et al*. 2007). Angiotensin‐II (AngII) induces constriction of efferent arterioles causing hypoperfusion of post‐glomerular peritubular capillaries, thus decreasing renal oxygen delivery (Calzavacca *et al*. 2015). Glomerular filtration rate (GFR) is usually maintained under such conditions (Treeck *et al*. 2002), so tubular sodium reabsorption, and thus renal oxygen consumption, is little changed. Consistent with this proposed mismatch between renal oxygen delivery and consumption, cortical $P_{O_{2}}$ could be relatively low in rats with AngII‐dependent hypertension. Insufficient availability of oxygen leads to cellular injury and loss of function (Mimura & Nangaku, 2010). Therefore AngII type 1 receptor (AT_1_R) blockade could be an interesting therapy for hypoxia. Accordingly, RAS inhibition has been found to improve cortical tissue oxygenation in anaesthetised rats both with (Welch *et al*. 2003; Manotham *et al*. 2004; Eckardt *et al*. 2005) and without (Norman *et al*. 2003) kidney disease. Although these studies were performed in an acute setting in animals under anaesthesia, their findings suggest that AngII can chronically have a negative impact on renal cortical oxygenation, which could potentially be a critical factor in the initiation and progression of CKD. Consistent with this concept, angiotensin‐converting enzyme inhibitors and AT_1_R blockade are still used as first‐line treatment in patients with CKD who do not require dialysis, and have been shown to improve survival (Qin *et al*. 2016).

The current understanding of the mechanisms underlying physiological regulation of kidney oxygenation, and the contribution of renal tissue hypoxia in the initiation and progression of renal disease, remains rudimentary (Evans *et al*. 2015). Recently we developed a technology that allows continuous and long‐term measurement of tissue $P_{O_{2}}$ in the kidney in unrestrained, conscious rats (Koeners *et al*. 2013). This allows investigation of renal oxygenation with high temporal resolution and without the confounding effects induced by anaesthesia. In the present study we utilised this technique to test the hypothesis that both exogenous and endogenous AngII promote renal hypoxia.

The specific aims of our current study were fourfold. In a first set of studies, we validated the use of telemetric measurement of renal cortical $P_{O_{2}}$ for more than 2 weeks in unrestrained rats and studied its physiological variation as well as its ability to respond to repeated periods of hypoxia and hyperoxia. In the second set of studies, in conscious rats, we compared acute responses of cortical $P_{O_{2}}$ to equi‐pressor doses (i.v. boluses and infusions) of AngII and the α_1_‐adrenoceptor agonist phenylephrine (PE). In the third set of studies, in anaesthetised rats, we examined the mechanisms underlying the differential sensitivity of renal cortical $P_{O_{2}}$ to AngII and PE, by also assessing their effects on renal oxygen delivery and consumption. In the fourth set of studies we determined in a cross‐over design whether prolonged activation (1 week) of endogenous renin could lead to renal cortical hypoxia in a rat model (the Cyp1A1Ren2 rat). This model is known to develop inducible AngII‐dependent hypertension and ultimately CKD when renin activation is prolonged for 4 weeks or more (Heijnen *et al*. 2011, 2013, 2014). We determined whether renal cortical hypoxia is an early event in this process and whether it can be reversed by AT_1_R blockade (Heijnen *et al*. 2013).

## Methods

### Ethical approval

All experiments were performed under license from the Animal Ethics Committee of University of Auckland (AEC R955) or the institutional Animal Care and Use Committee of Maastricht University (DEC2014‐012) and were all carried out according to the guidelines laid down by the New Zealand and Dutch Codes of Practice for the Care and Use of Animals for Scientific Purposes and complied with the policy and regulations of *The Journal of Physiology* (Grundy, 2015).

### System overview

The telemetry system, previously described by Russell *et al*. (2012), was adapted for renal tissue (Koeners *et al*. 2013, 2016). Briefly, the telemeter (TR57Y, Millar Inc., Houston, TX, USA) was equipped with a carbon paste electrode (CPE, 0.27 mm in diameter) for electrochemical detection of $P_{O_{2}}$. This CPE electrode was implanted in the rat kidney cortex, so that the tip of the electrode was approximately 2 mm below the cortical surface. Reference and auxiliary electrodes, made of silver wire (AG549511; Advent Research Materials, Suffolk, UK), were also implanted in the kidney. This maintained a potentiostat circuit with a potential of ‐650 mV on the CPE. The telemeter was placed in the abdomen of the rat and attached to the inner abdominal muscle layer. After a recovery period of 1 week, the rat's cage was placed on a receiver‐charging unit (SmartPad TR181, Millar Inc.), which received the data from, and recharged the battery of, the telemeter. This setup allowed renal cortical tissue $P_{O_{2}}$ to be measured continuously for several weeks at a frequency of 400 Hz (Wistar rats) or 5 Hz (Cyp1a1Ren2 rats).

### Animals

A total of 40 male Wistar rats (352 ± 13 g) and 8 male Cyp1A1Ren2 rats (377 ± 28 g) were used. Wistar rats were obtained from an internal breeding stock at the University of Auckland, originally derived from Charles River (USA). Cyp1A1Ren2 rats were obtained from an internal breeding stock at Maastricht University, originally derived from animals supplied by the Centre for Cardiovascular Science, University of Edinburgh, UK. These rats harbour a genetically inserted construct for the transcription of mRen2 preceded by a Cyp1a1 promoter on the Y‐chromosome. Cyp1a1 can be induced by adding an aryl hydrocarbon agonist, such as indole‐3‐carbinol (I3C), to the diet. This leads to an increase in (pro)renin levels and subsequently a blood pressure increase (Heijnen *et al*. 2013). All animals were housed with a 12 h light–dark cycle at a temperature of 20–22°C and allowed free access to water and standard rat chow *ad libitum*. In Cyp1A1Ren2 rats, I3C was mixed through the diet to achieve concentrations of 0.3 or 0.6% w/w (Heijnen *et al*. 2013). This diet was administered to activate the RAS in these transgenic rats. After the experimental period, all rats were killed by intraperitoneal injection of an overdose of sodium pentobarbitone (>200 mg ml^−1^, Provet NZ PTY Ltd, Auckland, NZ) and postmortem $P_{O_{2}}$ values were determined for off‐set correction of individual $P_{O_{2}}$ recordings.

### Validation of the system in the rat kidney cortex

#### Surgery/implantation

Telemeters were prepared and surgery was performed as previously described (Koeners *et al*. 2013, 2016). In summary, telemeters were sterilised in a 2% w/v glutaraldehyde solution for at least 4 h and rinsed thoroughly with sterile 0.9% w/v NaCl solution before implantation. Male Wistar rats (*n = *15) were anaesthetised with 5% v/v isoflurane in an induction box and maintained at 2–2.5% v/v isoflurane via a mask on a heated operating table. Rats were pre‐medicated with enrofloxacin (5 μg kg^−1^ s.c., Baytrill, SVS Vet Supplies Ltd, Auckland, NZ) and buprenorphine (30 μg kg^−1^ s.c., Temgesic, Reckitt Benckiser, Auckland, NZ, or ASTfarma B.V., Ouderwater, Netherlands). Under sterile conditions, the left kidney and aorta were exposed by laparotomy. The cables connecting the electrodes and telemeter were secured by suturing them on the adventitia of the abdominal aorta or dorsal muscles adjacent to the spine near the left kidney. After pre‐puncturing the kidney with a 30 gauge needle, the reference electrode and CPE, both with a right‐angled bend 2 mm from the tip, were inserted in the kidney cortex and secured in place with tissue glue (Histoacryl, 1050044 B. Braun, Tuttlingen, Germany) approximately 1 mm apart from each other, while the auxiliary electrode was affixed onto the kidney surface. With the telemeter placed and secured in the abdomen, the abdomen was closed with sutures and the rat was placed on a heated pad for at least 12 h to recover. Post‐operative analgesia was administered (buprenorphine, 3 μg (100 g^−1^) every 8–14 h for up to 3 days or as required).

#### Cortical $P_{O_{2}}$ by CPE‐telemetry for 19 days

**Figure 1:**
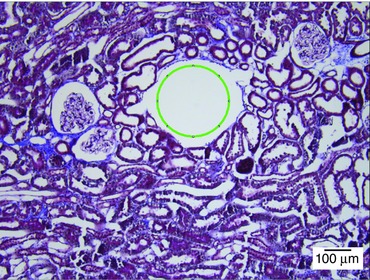
**Representative section of the renal cortex, 3 weeks after implantation of the electrode, stained with Masson's trichrome** Fibrotic tissue is represented by blue, the nuclei by dark purple, and other cellular compartments by red. Electrode diameter and position are indicated by a green circle. Photomicrograph at ×10 magnification. [Colour figure can be viewed at wileyonlinelibrary.com]

Renal cortical $P_{O_{2}}$ was continuously measured in nine Wistar rats for 19 days. The coefficient of variation (CV) of the $P_{O_{2}}$ signal was assessed in each rat over the last 14 days (days 6–19) for each recording. CV was calculated in percentage as (standard deviation (individual means for a specific time interval))/(all means for a specific time interval). The three specific time intervals we used were: days 6–19, 24 h, and 5 min. Kidneys were processed for assessment of tissue damage and scarring around the implanted electrode tip by Masson's trichrome staining. As found previously in the renal medulla (Koeners *et al*. 2013), there was little or no scarring associated with implantation of the electrodes in the cortex (Fig. 1).

#### Responses to altered inspired oxygen content

During the 19 days after implantation of the telemeter, Wistar rats underwent repetitive hypoxia/hyperoxia trials to determine the reproducibility of responses of the system to changes in cortical tissue $P_{O_{2}}$. Details of this protocol were described previously (Koeners *et al*. 2013). Briefly, at 3–4 day intervals the rat's home cage was placed in a custom‐made Perspex chamber (65 cm x 45 cm x 35 cm) which was filled with hypoxic air (10% oxygen) or hyperoxic air (100% oxygen) for 30 min periods (5 l min^−1^). The O_2_ concentration in the sealed box was measured using a gas analyser (ADInstruments). The $P_{O_{2}}$ during the final 5 min of each trial was compared with that recorded while rats breathed room air (21% oxygen). Hypoxic and hyperoxic conditions were presented in random order and each challenge was preceded and followed by a 30 min period in which the rat was exposed to room air.

### Effects of angiotensin II and phenylephrine on renal cortical oxygen tension

#### Conscious rats

Male Wistar rats (*n = *7, 333 ± 15 g) were instrumented with CPE‐telemeters as described above. Another group of male Wistar rats (*n = *6, 339 ± 11 g) was instrumented with blood pressure telemeters (TRM56SP, Millar Inc.) and a blood pressure catheter was introduced and fixed into the abdominal aorta as described previously (Lau *et al*. 2013). During the same surgical procedure both groups were equipped with a catheter placed in the left femoral vein. This catheter was tunnelled subcutaneously so that it exited between the shoulder blades. The catheters were made of heparin coated tubing (Micro‐Renathane model MRE 33 connected to model MRE 40 tubes, 3 cm and 16 cm in length, respectively). Following surgery, a 5 day recovery period was allowed before experimental manipulations commenced. Stable recording was achieved when within‐animal coefficient of variation (CV) of the $P_{O_{2}}$ signal over 24 h was less than 31 ± 6% for 2 days. During the AngII (Auspep Pty Ltd, Victoria, Australia) and PE (Sigma, USA) intervention, rats were placed in a custom‐made Perspex chamber (25 cm x 45 cm x 35 cm) and acclimatised for 30 min while their femoral vein catheter was connected to tubing leading out of the box. Baseline $P_{O_{2}}$ was defined as the average during the final 10 min of this period. To avoid acute (seconds) desensitisation to AngII (Guo *et al*. 2001), i.v. bolus injections (5 μg ml^−1^ kg^–1^) were given at 0, 5, 10, 15, and 30 s, in volumes of 1, 2, 7, 20, and 70 μl, in order to give cumulative doses of 5, 15, 50, 150, and 500 ng kg^–1^, respectively. Averages of the last 2 s before the next step were used to calculate $P_{O_{2}}$ and mean arterial pressure (MAP). The dose range was chosen based on previous experience (Nelissen‐Vrancken *et al*. 1992). On the same day, after a 60–120 min recovery period, bolus doses of PE of 20, 200, 500, 1000 and 1500 ng kg^–1^ were injected in volumes of 100 μl at 5 min intervals. These doses were chosen to produce similar changes in MAP as those evoked by AngII. Averages from 30 to 40 s after the bolus were used to calculate $P_{O_{2}}$. The entire protocol was repeated 2–3 days later, except that the order of the treatments was reversed (i.e. PE was administered before AngII).

Subsequently, responses to 10 min infusions of AngII (150 ng min^−1^ kg^–1^, the second highest dose given in the bolus injection) and PE (7.2 μg min ^−1^ kg^–1^, chosen to produce similar changes in MAP as those evoked by AngII) were determined. The $P_{O_{2}}$ and MAP were averaged over the final 60 s of each infusion. This protocol was repeated in a reverse order (i.e. PE first, then AngII) 2–3 days later.

#### Anaesthetised rats

In terminal experiments, MAP and RBF (transit‐time ultrasound) were determined in male Wistar rats (*n = *25, 361 ± 11 g) as described previously (Koeners *et al*. 2007). Briefly, all rats were anaesthetised with 5% v/v isoflurane, intubated and then artificially ventilated (model 680; Harvard Apparatus, Holliston, MA, USA) with 2–2.5% v/v isoflurane in 100% O_2_ and a tidal volume of ∼3–4 ml and frequency of ∼70 breaths min^−1^. Catheters were placed in the jugular and femoral veins for infusions. The femoral artery was catheterised to measure MAP and collect blood. The left ureter and bladder were cannulated in order to collect urine. The infusion solution contained inulin to calculate GFR by inulin clearance as described previously (Racasan *et al*. 2003). The sodium content of urine and plasma samples was determined by flame photometry (IL 543, Instrument Laboratory, Lexington, MA, USA). Cortical $P_{O_{2}}$ was determined using the telemeter and electrodes, implanted acutely, as described previously (Koeners *et al*. 2013). During the control period, urine was collected over two 10 min periods and arterial blood samples (0.2–0.3 ml) were collected before and after the entire 20 min period. After these control clearance periods, an intravenous infusion of either saline (*n* = 9), AngII (100–140 ng min ^–1^ kg^–1^, *n* = 8) or PE (3.8 μg min ^−1^ kg^–1^, *n* = 8) commenced. Once arterial blood pressure and cortical $P_{O_{2}}$ had stabilised at the new level, urine and blood were collected over two 10 min clearance periods. Arterial (via femoral catheter) and renal venous blood (via renal vein) samples were then obtained for oximetry at the end of the experiment (i.e. during infusion of saline, AngII or PE). Blood oxygen content (O_2_ct) was calculated as O_2_ct = ([haemoglobin (g l^−1^)] × oxygen saturation × 1.34) + (blood $P_{O_{2}}$ × 0.003). Renal oxygen delivery ($D_{O_{2}}$) was calculated as the product of RBF and arterial O_2_ct, while renal oxygen consumption ($Q_{O_{2}}$) was calculated as the product of RBF and the difference between arterial and renal venous blood oxygen content (Papazova *et al*. 2015).

#### Sub‐acute activation of the endogenous renin–angiotensin system

Male transgenic Cyp1a1Ren2 rats (*n* = 8) were instrumented with CPE‐telemeters in the renal cortex as described above. In a cross‐over design, after a 7 day recovery period, four rats were exposed to a 0.3% w/w I3C (Sigma‐Aldrich, St Louis, MO, USA) containing diet for 1 week. The genetic construct in these animals, which is activated by I3C, dose‐dependently stimulates hepatic production of mouse renin (Mullins *et al*. 1990) and causes AngII‐dependent glomerular sclerosis, tubular atrophy, and renal parenchymal inflammation after 4 weeks, accompanied by hypertension (Kantachuvesiri *et al*. 2001; Heijnen *et al*. 2013). The other four rats remained on the normal (control) diet. During the second week, diets were switched between groups. In week 4 both groups were fed a 0.6% w/w I3C containing diet. After 2–4 days of 0.6% I3C feeding, rats received a subcutaneous injection of the angiotensin receptor antagonist losartan (MSD, Oss, the Netherlands, 30 mg kg^−1^, *n* = 7). Cortical $P_{O_{2}}$ was recorded continuously during the experiment. To determine the effect of the diets, all data were averaged over 3 h epochs. Baseline $P_{O_{2}}$ (= 100%) was set as the group average $P_{O_{2}}$ value calculated over 6 h before commencing the dietary intervention. To isolate the effects of AT_1_R blockade, data were averaged for 5 min periods and a new baseline (= 100%) was calculated as the group average $P_{O_{2}}$ value over 15 min before injection. Baseline $P_{O_{2}}$ was calculated from the group $P_{O_{2}}$ averages, instead of the individual $P_{O_{2}}$ averages, in order to demonstrate the between‐animal/probe variation.

#### Analysis of $P_{O_{2}}$ data

Raw $P_{O_{2}}$ data were filtered with a 25 Hz low‐pass filter (data from Wistar rats) and artifacts were removed when the 1st order derivative exceeded a threshold of 5–300 nA s^−1^ (Wistar and Cyp1a1Ren2 rats), as described previously (Koeners *et al*. 2013).

All $P_{O_{2}}$ data are expressed as relative values from individual baseline recordings unless stated otherwise.

### Statistical analysis

Data are expressed as means ± SEM. They were subjected to Levene's mean test (to test equality of variance) and repeated measures analysis of variance (ANOVA), or one‐ or two‐way ANOVA. To protect against increased risk of type 1 error arising from the use of multiple comparisons, either Tukey's test (when all possible comparisons were made) or Dunnett's test (when multiple treatments were compared to a control condition) was applied. Statistics were performed using SigmaPlot software (Systat Software Inc, California, CA, USA). Differences were considered statistically significant if two‐sided *P ≤ *0.05 and are presented as unequal lower case letters within the figures.

## Results

### Validation of chronic telemetric measurement of tissue $P_{O_{2}}$ in the rat kidney cortex

**Figure 2:**
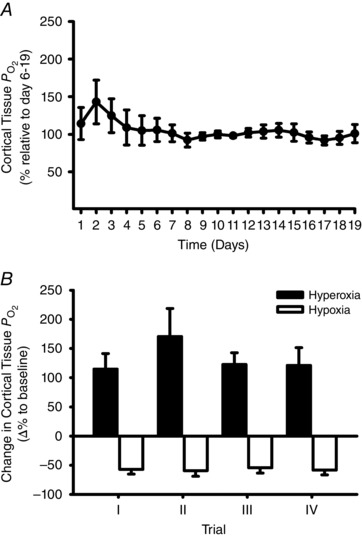
**Validation of long‐term measurement of cortical tissue** $P{\;}_{O_{2}}$ *A*, mean daily cortical tissue $P_{O_{2}}$ over a 19 day period, expressed as a percentage of the average values on days 6–19 across all rats (*n = *9). *B*, mean change in cortical tissue $P_{O_{2}}$ in response to hyperoxia (filled bars) and hypoxia (open bars) in all rats (*n = *9), compared to baseline values assessed during normoxia (room air, 21% oxygen). I–IV represent the successive trials performed at 3–4 day intervals. Data are expressed as between‐rat means ± SEM.

Renal cortical tissue $P_{O_{2}}$ was labile during the first 5 days after implantation, but thereafter remained relatively stable for the 14 day observation period (Fig. 2 *A*). To quantify the stability of the $P_{O_{2}}$ signal, the average within‐animal CV of the $P_{O_{2}}$ signal was calculated over three specific time intervals (days 6–19, 24 h and 5 min) over the entire period of the last 14 days (*n = *9). The average mean CV derived from days 6–19 was 22.5 ± 3.8%, that from the 24 h interval was 21.8 ± 2.1% and that from the 5 min interval was 5.1 ± 0.3%.

The responsiveness and reproducibility of telemetric recordings of cortical $P_{O_{2}}$ were tested *in vivo* by hypoxia/hyperoxia trials (Fig. 2 *B*). In all trials (4 trials in each of 9 animals) hyperoxia substantially increased cortical $P_{O_{2}}$, by 132 ± 31% with a mean CV derived from a 1 min time interval of 4.6 ± 0.8%. Cortical $P_{O_{2}}$ decreased during hypoxia in all trials, with a mean reduction of 57 ± 9% and a mean CV derived from a 1 min time interval of 5.9 ± 1.6%.

### Effects of exogenous angiotensin II and phenylephrine on cortical oxygenation

#### Acute vasoconstrictor‐specific responses

**Figure 3:**
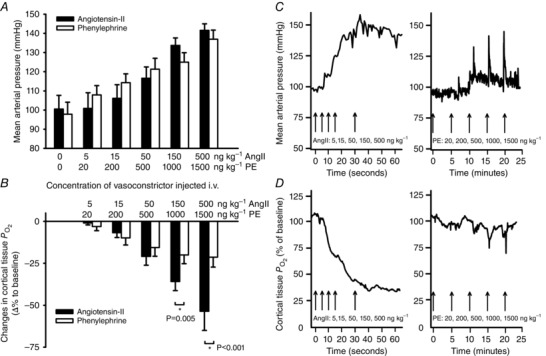
**Responses of arterial pressure and cortical tissue** $P{\;}_{O_{2}}$ **to intravenous boluses of angiotensin‐II and phenylephrine in conscious rats** *A*, peak mean arterial pressure during intravenous (i.v.) bolus injections of increasing doses of angiotensin‐II (AngII, filled bars) and phenylephrine (PE, open bars) titrated to produce equi‐pressor effects (*n = *6). *B*, mean changes of cortical $P_{O_{2}}$ in response to i.v. injections of the same increasing doses of AngII (filled bars) and PE (open bars) in a separate cohort of rats (*n = *7). *C* and *D*, original individual tracings of mean arterial pressure and cortical $P_{O_{2}}$, respectively, during i.v. injection of AngII and PE, recorded in a representative rat. Data in panels *A* and *B* are shown as means ± SEM. Data were subjected to RM ANOVA (equal variance test: *A*, passed, *P* = 0,742; *B*, passed, *P* = 0,223) followed by Tukey's *post hoc* test. Differences were considered statistically significant if two‐tailed *P ≤ *0.05(^*^).

In conscious rats AngII and PE were administered as i.v. bolus injections titrated to result in pressor responses of similar magnitude, as assessed by telemetric measurement of arterial pressure (Fig. 3 *A*). Renal cortical $P_{O_{2}}$ was reduced by both PE and AngII (Fig. 3 *B*). At higher doses, the effects of AngII on $P_{O_{2}}$ in the renal cortex were greater than those of PE. For example, a dose of 500 ng kg^–1^ AngII decreased $P_{O_{2}}$ by 54 ± 14%, whereas $P_{O_{2}}$ was decreased only by 21 ± 7% at an equi‐pressor dose of 1500 ng kg^–1^ PE. Responses of MAP and cortical $P_{O_{2}}$ to AngII and PE developed within seconds of the injection (Fig. 3 *C* and *D*).

**Figure 4:**
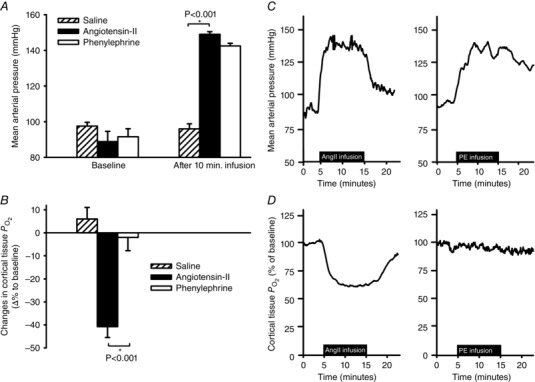
**Changes in mean arterial pressure and cortical tissue** $P{\;}_{O_{2}}$ **during intravenous infusions of angiotensin‐II and phenylephrine in conscious rats** *A*, mean arterial pressure at baseline and during a 10 min intravenous (i.v.) infusion of saline (hatched bars), angiotensin‐II (AngII, filled bars) and phenylephrine (PE, open bars) (*n = *5). *B*, mean changes of cortical $P_{O_{2}}$ in response to 10 min. i.v. infusion of AngII (filled bars) and PE (open bars, equal‐pressure doses) (*n = *6). *C* and *D*, original tracings of mean arterial pressure and cortical $P_{O_{2}}$, respectively, before, during and after i.v. infusion of AngII or PE, recorded in a representative rat. Data from panels *A* and *B* are shown as means ± SEM. Data was subjected to one‐way ANOVA followed by Tukey's *post hoc* test. Differences were considered statistically significant if *P ≤ *0.05(^*^)

The differential effects of AngII and PE on cortical oxygenation were more pronounced when these agents were administered as short‐term infusions than when administered as boluses. Cortical $P_{O_{2}}$ fell by 41 ± 5% during an infusion of AngII at a dose of 150 ng min^−1^ kg^–1^ but was not significantly altered by PE at a dose of 7.2 μg min ^−1^ kg^–1^, (−2 ± 6% change) (Fig. 4 *B*). These infusions of AngII and PE resulted in similar increases in MAP (Fig. 4 *A*). Infusion of AngII caused a prolonged effect, which was normalised only when the infusion was stopped. Original tracings during AngII and PE infusion in a representative rat are shown in Fig. 4 *C* and *D*.

### Effects of exogenous angiotensin II and phenylephrine on renal blood flow and oxygenation in anaesthetised rats

**Figure 5:**
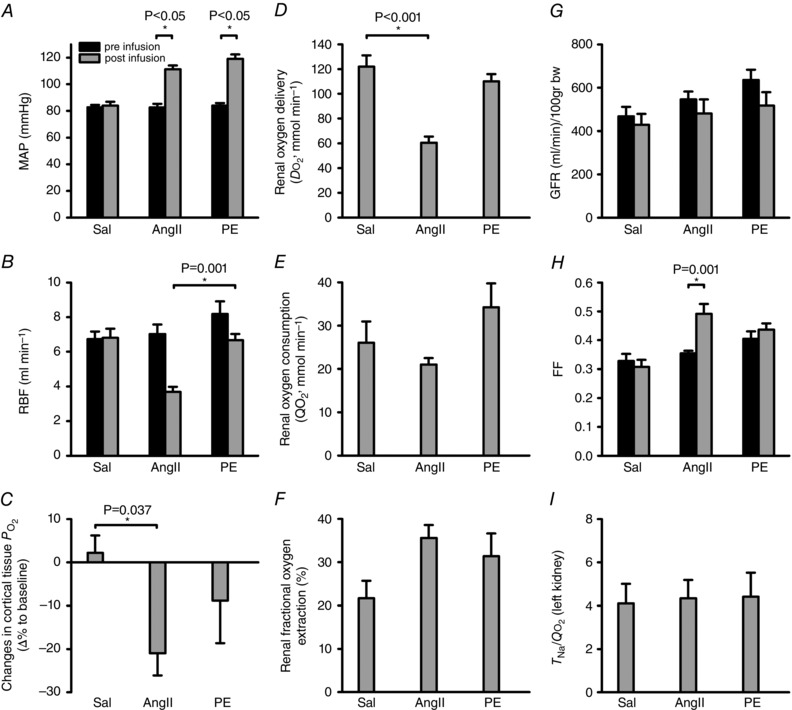
**Responses to intravenous infusions of angiotensin‐II and phenylephrine in anaesthetised rats** Responses of mean arterial pressure (*A*) and renal blood flow (*B*) before (black bar) infusion or after (grey bar) 10 min intravenous infusions, and changes in cortical tissue $P_{O_{2}}$ (*C*) after infusions of saline (Sal, *n = *9), angiotensin‐II (AngII, *n = *7) and phenylephrine (PE, *n = *8). Renal oxygen delivery (*D*), renal oxygen consumption (*E*), renal fractional oxygen extraction (*F*) after 10 min intravenous infusions of Sal (*n = *7), AngII (*n = *7) and PE (*n = *5). Glomerular filtration rate (GFR; *G*), filtration fraction (FF; *H*), and *T*_Na_/$Q_{O_{2}}$ (*I*) after 10 min intravenous infusions of Sal (*n = *7), AngII (*n = *7) and PE (*n = *5). Data shown as means ± SEM and subjected to two (*A*, *B*, *G*, *H*)‐ or one (*C*, *D*, *E*, *F*, *I*)‐way ANOVA followed by Tukey's *post hoc* test. Differences were considered statistically significant if two‐tailed *P ≤ *0.05(^*^).

In anaesthetised rats, cortical $P_{O_{2}}$ decreased by 21 ± 5% during infusion of AngII. PE infusion did not change cortical $P_{O_{2}}$ significantly −9 ± 10% change, Fig. 5 *C*). The two infusions resulted in similar increases in MAP (Table 1, Fig. 5 *A*). However, the magnitude of the change in cortical $P_{O_{2}}$ associated with infusion of AngII and PE did not differ significantly. RBF decreased more (3.3 ± 0.3 ml min^−1^) during infusion of AngII than during infusion of PE (1.5 ± 0.4 ml min^−1^, Fig. 5 *B*). Infusion of the saline vehicle resulted in little or no change in cortical $P_{O_{2}}$, MAP or RBF.

**Table 1:** Haemodynamic parameters before and during vasoconstrictor infusions in anaesthetised rats

	**Baseline**	**Saline**	**Baseline**	**AngII**	**Baseline**	**PE**
			
*n*	9		7		7	
MAP (mmHg)	83 ± 2	84 ± 3	83 ± 3	111 ± 3*#	84 ± 2	119 ± 3*#
RBF (ml min^–1^)	6.7 ± 0.4	6.8 ± 0.5	7.0 ± 0.6	3.7 ± 0.3*#	8.2 ± 0.7	6.7 ± 0.4*
Cortical $P_{O_{2}}$ (Δ%)	—	2.2 ± 4.0	—	−21.0 ± 5.1#	—	−8.8 ± 9.8
FF	0.33 ± 0.02	0.31 ± 0.02	0.35 ± 0.01	0.49± 0.03*#	0.41 ± 0.03	0.44 ± 0.03

Data are expressed as means ± SEM. Data were subjected to one‐way (Cortical $P_{O_{2}}$) or two‐way (MAP, RBF, FF) ANOVA followed by Tukey's *post hoc* test. Differences were considered statistically significant if two‐tailed *P* ≤ 0.05. ^*^Significant difference *versus* baseline within the same group; ^#^significant difference *versus* post‐infusion of saline. MAP = mean arterial pressure; RBF = renal blood flow; FF = filtration fraction.

Compared to rats receiving an infusion of the saline vehicle, renal oxygen delivery was significantly more reduced (50 ± 4%) in rats receiving AngII than in rats receiving PE (10 ± 5%, *P* < 0.05, Fig. 5 *D*). However, the other measures of whole kidney oxygenation, renal oxygen consumption (Fig. 5 *E*) and fractional extraction (Fig. 5 *F*) did not differ significantly between rats treated with saline, AngII, or PE. GFR was not significantly altered during any infusion (Fig. 5 *G*). However, filtration fraction increased by 39 ± 10% during infusion of AngII (Fig. 5 *H*) but did not change significantly during infusion of saline or PE. No significant differences in the ratio between tubular sodium reabsorption and $Q_{O_{2}}$ (*T*_Na_/$Q_{O_{2}}$) were found between the three treatment groups (Fig. 5 *I*).

### Effects of activation of the endogenous renin–angiotensin system on cortical oxygenation

**Figure 6:**
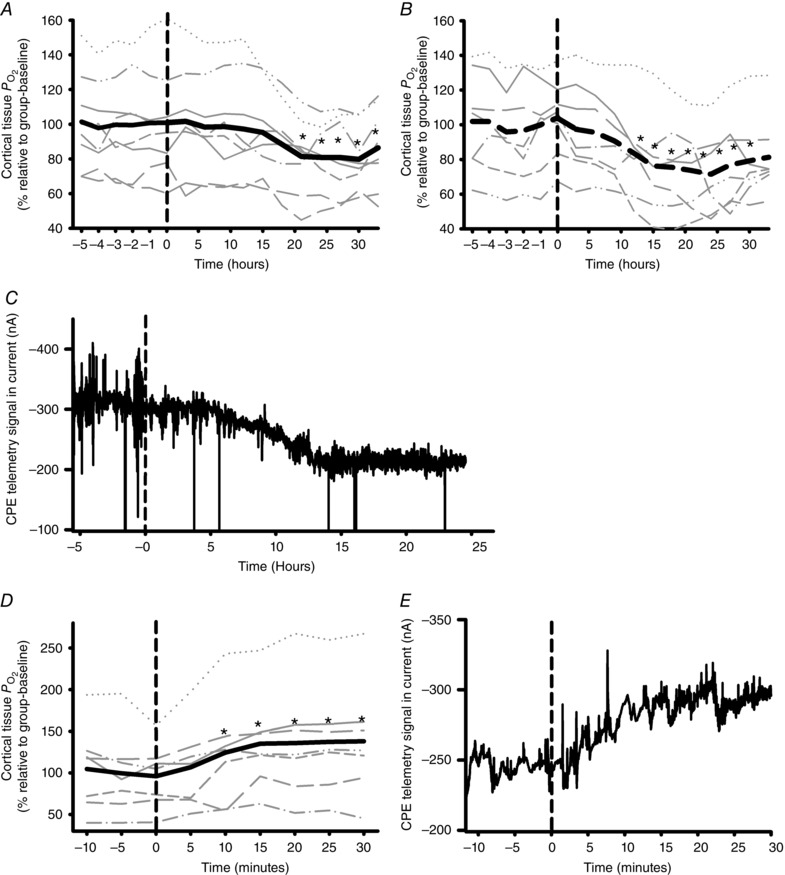
**Effects of activation of the endogenous renin–angiotensin system** Mean cortical tissue $P_{O_{2}}$ is expressed as a percentage of the average values during the 6 h before commencing feeding with a diet containing either 0.3% w/w (*A*, *n = *8) or 0.6% w/w (*B*, *n = *7) indole‐3‐carbinol (I3C). Grey lines represent data for individual rats, while the thick black line represents the between‐rat mean. *C*, original tracing, obtained with 5 Hz sample frequency, of the current recorded in a representative rat, before and during feeding with 0.6% I3C (expressed as nA). The dashed line indicates commencement of the I3C diet. CPE, carbon paste electrode. Data was subjected to RM ANOVA (equal variance test: *A*, passed, *P* = 0,130; *B*, passed, *P* = 0,901) followed by Dunnett's *post hoc* test. Differences were considered statistically significant if two‐tailed *P* ≤ 0.05(^*^) *vs*. baseline. Effects of blockade of the endogenous renin–angiotensin system. *D*, mean cortical tissue $P_{O_{2}}$ before and after subcutaneous injection of losartan during feeding with 0.6% w/w indole‐3‐carbinol (I3C). $P_{O_{2}}$ is expressed as a percentage of the average values during the 10 min period prior to injection of losartan. Grey lines represent data for individual rats, while the thick black line represents the between‐rat means. *E*, original tracing, obtained with 5 Hz sample frequency, of the current recorded in a representative rat, expressed as nA, before and after subcutaneous injection of losartan. The dashed line indicates the moment of losartan injection. CPE, carbon paste electrode. Data were subjected to RM ANOVA (equal variance test: *A*, passed, *P* = 0,163) followed by Dunnett's *post hoc* test. Differences were considered statistically significant if two‐tailed *P* ≤ 0.05(^*^) *vs*. baseline.

When 0.3% I3C was added to the food of the Cyp1a2Ren2 transgenic rats, renal cortical $P_{O_{2}}$ gradually fell over the subsequent 30 h. A nadir was reached 25 ± 4 h after the presentation of the diet (Fig. 6 *A*) with cortical $P_{O_{2}}$ being 20 ± 6% below its baseline value. During the challenge with 0.6% I3C the nadir in cortical $P_{O_{2}}$, a reduction of 29 ± 9% below baseline, was observed 23 ± 10 h after the presentation of the diet (Fig. 6 *B*).

Blockade of the AT_1_R in Cyp1a1Ren2 transgenic rats fed 0.6% I3C resulted in a rapid increase of cortical $P_{O_{2}}$. Peak responses were reached 14 ± 3 min after s.c. administration of losartan, where cortical $P_{O_{2}}$ was 37 ± 7% higher than before injection (Fig. 6 *D*). Original tracings to illustrate the dynamics of the responses to the I3C diet and losartan are shown in Fig. 6 *C* and 6 *E*.

## Discussion

The results of this study show that, in conscious unrestrained rats, renal cortical oxygen tension is decreased by stimulation of the AT_1_R by both exogenous and endogenous AngII. Using devices for telemetric measurement of tissue $P_{O_{2}}$, we show for the first time in conscious unrestrained rats that acute renal cortical hypoxia induced by exogenous AngII is (1) greater than that evoked by equi‐pressor doses of phenylephrine; and (2) is mainly caused by reduced RBF rather than by increased oxygen consumption. We also show that relative renal cortical hypoxia (3) occurs relatively fast (within 24 h) after activation of endogenous AngII, well before histological manifestation of renal disease, and (4) can be rapidly (within minutes) reversed by an AT_1_ receptor antagonist. These new data are consistent with the hypothesis that AngII‐induced renal cortical hypoxia is a causal factor in the aetiology of renal disease rather than a consequence of other pathological processes.

### Validation of chronic telemetric measurement of tissue $P_{O_{2}}$ in the renal cortex

The ability to assess renal tissue $P_{O_{2}}$ in unrestrained rats over prolonged periods provides a new tool to investigate the role of renal tissue hypoxia in the pathogenesis of kidney disease. Previously, we demonstrated that the device utilised in the current study can be employed to assess tissue oxygenation in the renal medulla of the rat (Koeners *et al*. 2013). Now we extend these studies by demonstrating that this method is also applicable in the renal cortex. We validated the methodology by showing that measurements of cortical $P_{O_{2}}$ were stable over a period of 6–19 days after implantation of the CPE telemeter. Long‐ and short‐term coefficients of variation were 22% and 5%. These are comparable to those reported for blood pressure or heart rate in rats (Teerlink & Clozel, 1993; Jiang *et al*. 2016). Secondly we showed that the method could reproducibly detect acute changes in cortical oxygenation induced by systemic hyperoxia and hypoxia. Variations to these repeated challenges were as low as 5–6%. These data demonstrate that the technique is able to reproducibly detect changes in renal tissue oxygenation induced by systemic challenges. This provides strong evidence that the system is reliable for long‐term monitoring of renal cortical oxygenation in the rat.

### Effects of exogenous angiotensin II and phenylephrine on cortical oxygenation in conscious rats

We then used this method to assess the influence of exogenous AngII on cortical $P_{O_{2}}$. Cortical tissue $P_{O_{2}}$ was reduced immediately by intravenous administration of AngII. We can be confident that the reduction in cortical $P_{O_{2}}$ induced by AngII is not a consequence of its pressor effect, because equi‐pressor doses of PE had a much lower effect on tissue $P_{O_{2}}$, irrespective of whether the agents were administered as boluses or infusions. It seems likely that the differential effects of AngII and PE on renal oxygenation reflect their disparate actions at the level of the renal vasculature.

### Effects of exogenous angiotensin II and phenylephrine on renal blood flow and oxygenation in anaesthetised rats

The rapidity with which cortical tissue $P_{O_{2}}$ fell in response to administration of AngII, either as a bolus or infusion, supports the idea that this effect is driven predominantly by the impact of AngII on RBF (Polichnowski *et al*. 2013), and thus renal oxygen delivery, rather than on kidney oxygen consumption (Friederich‐Persson *et al*. 2014). Indeed it is well known that AngII decreases renal blood flow (Hollenberg *et al*. 1976; Yamamoto *et al*. 2001; Kobori *et al*. 2007). To verify this, we performed studies in anaesthetised rats in which we could quantify renal oxygenation and haemodynamics. AngII reduced cortical $P_{O_{2}}$, RBF and $D_{O_{2}}$ more than PE did. We could not detect effects of AngII or PE on $Q_{O_{2}}$, renal fractional oxygen extraction or *T*_Na_/$Q_{O_{2}}$. The present data contrast with those from a study by Welch *et al*. in which a decreased *T*_Na_/$Q_{O_{2}}$ was observed. However, the latter studies were done after prolonged (2 week) infusion of AngII (Welch *et al*. 2005). Critically, long‐term effects of this potent peptide might be quite different from those evoked by the short‐term bolus injections or 10 min infusions that we used. Hence, in the present study, the more pronounced reduction in cortical $P_{O_{2}}$ in response to AngII compared to PE is likely to be due to their differential effects on oxygen delivery to the renal cortex. In awake sheep, AngII did not significantly alter renal cortical $P_{O_{2}}$ when RBF and renal oxygen consumption were reduced by 21% and 18%, respectively (Calzavacca *et al*. 2015). Similar results were observed in anaesthetised rabbits (Evans *et al*. 2011). Thus, it appears that a threshold in the reduction in RBF (>30%) must be exceeded before cortical hypoxia occurs. In addition, total RBF and $D_{O_{2}}$ do not take into account regional variations in blood flow, oxygen delivery/consumption, and potentially also changes in counter‐current shunting of oxygen (Evans *et al*. 2008). Together this probably explains why PE did not significantly reduce cortical $P_{O_{2}}$. Furthermore, renal resistance was reduced and RBF increased after a non‐hypotensive dose of captopril in healthy conscious rats (Nelissen‐Vrancken *et al*. 1992), suggesting, although not decisively, that RAS inhibition could increase renal tissue $P_{O_{2}}$ by increasing $D_{O_{2}}$. The maintenance of ${\dot{Q}}_{O_{2}}$ during infusion of AngII is probably due to the maintenance of GFR (as reflected in increased FF), and thus the filtered load of sodium. Consistent with this proposition, van der Bel *et al* recently showed a strong association of reduced cortical oxygenation (as assessed by blood oxygen level‐dependent magnetic resonance imaging) with increased filtration fraction during intravenous infusion of angiotensin II in man (van der Bel *et al*. 2016).

Differences between the systemic and renal effects of AngII and PE were greater in conscious than in anaesthetised animals. Potentially, in conscious animals, the ability of AngII to act presynaptically to facilitate sympathetic neurotransmission (Nap *et al*. 2002) could be greater than that in anaesthetised animals. Isoflurane and other inhalation anaesthetics tend to inhibit the activity of the sympathetic nervous system (Pac‐Soo *et al*. 2000). They can also blunt the response of the vasculature to AngII and increase the sensitivity of the vasculature to α‐adrenoceptor activation (Yu *et al*. 2004; Bussey *et al*. 2014). These considerations emphasise the importance of avoiding the confounding effects of anaesthesia in studies of the physiological control of renal oxygenation. Hence, a definitive conclusion regarding the relative roles of changes in oxygen delivery *versus* oxygen consumption in AngII‐induced cortical hypoxia can only be made by simultaneous *in vivo* measurement of these variables in conscious animals. This is not (yet) possible in rats.

### Activation of the endogenous renin–angiotensin system on cortical oxygenation

Activation of the endogenous renin–angiotensin system reduced cortical $P_{O_{2}}$. The time course of the effect in conscious Cyp1a1Ren2 transgenic rats was consistent with previous studies in this model that have documented activation of the RAS. For example, arterial pressure was found to have increased by approximately 12 h after administration of I3C by gastric gavage, while pro‐renin levels were found to have increased within 6 h (Kantachuvesiri *et al*. 2001). In the current study we found a statistically significant decrease in $P_{O_{2}}$ after 15 h of dietary exposure to I3C. The slower time course of effects in our study can be attributable to the fact that I3C was delivered via the food rather than by gastric gavage. The immediate reversal of hypoxia by AT_1_R blockade indicates that the effects of I3C feeding on cortical $P_{O_{2}}$ were mediated directly through activation of AT_1_ receptors and exclude other components of the RAS (e.g. aldosterone). This is in line with the findings of Norman *et al*., who observed increased cortical $P_{O_{2}}$ coinciding with increased RBF and microvascular flow after acute AT_1_R blockade in healthy (anaesthetised) rats (Norman *et al*. 2003). Prolonged activation of the RAS in Cyp1a1Ren2 rats causes hypertension, increased renal vascular resistance (as measured by flow probe), glomerulosclerosis and tubulointerstitial inflammation (Heijnen *et al*. 2011, 2013). In addition, in Cyp1a1Ren2 rats, the kidney seems to be more at risk of development of injury than the heart, which showed only relatively mild maladaptation (i.e. mild and reversible myocardial concentric hypertrophic remodelling) despite the fulminant hypertension (Heijnen *et al*. 2014). Our current findings indicate that renal hypoxia in this model is likely to be driven by renal vasoconstriction, and precedes development of overt renal pathology, which is not observed until 4 weeks after initiation of I3C treatment (Kantachuvesiri *et al*. 2001; Heijnen *et al*. 2013). Thus, renal hypoxia is more likely to be a stimulus that contributes to the pathological process rather than just a consequence of pathology.

To the best of our knowledge, our findings are the first to provide direct evidence that endogenous AngII can reduce cortical tissue $P_{O_{2}}$ in conscious, unrestrained rats, through activation of AT_1_R. Our findings are consistent with those of others, of the effects of AT_1_R blockade in anaesthetised rats (Norman *et al*. 2003; Manotham *et al*. 2004). These findings pave the way for detailed investigation of the temporal relationships between renal cortical hypoxia and renal pathology in chronic kidney disease and acute kidney injury.

Three important limitations of our study must be acknowledged. Firstly, we are unable to measure tissue $P_{O_{2}}$ in more than one place using our current telemetric approach. This precludes us from characterising the spatial relationships between tissue hypoxia and renal pathology using this method. Secondly, we are unable to quantify renal oxygen delivery and consumption in un‐anaesthetised rats. Thus, we had no alternative than to measure these variables in groups of anaesthetised and ventilated rats. Nevertheless, the responses of cortical tissue $P_{O_{2}}$ to AngII in anaesthetised rats were similar to those in conscious rats. Thus, we can be confident that our observations in rats under anaesthesia are relevant to the interpretation of our studies in conscious animals. Thirdly, as discussed previously (Koeners *et al*. 2013) we remain cautious about using absolute $P_{O_{2}}$ values due to discrepancies between the calibration parameters before *versus* calibration after implantation, and the presence of an additional zero offset under *in vivo* conditions. We consequently used relative values in our analysis instead of absolute concentration. This offset places restraints on study design, because it makes direct between‐animal comparisons problematic. However, it does not limit the use of within‐animal experimental designs in which changes in $P_{O_{2}}$ are determined from a baseline established before the intervention, as was done in this study.

In conclusion, our current findings indicate that it is feasible to investigate tissue oxygenation in the renal cortex by telemetry. We found that exogenous AngII rapidly and markedly reduced cortical $P_{O_{2}}$ in conscious rats. Our data suggest that the effect is mainly due to decreased tissue oxygen delivery as a consequence of cortical vasoconstriction and reduced RBF. Activation of the endogenous RAS in the Cyp1a1Ren2 transgenic rat resulted in reduced cortical tissue $P_{O_{2}}$ which was rapidly reversed by AT_1_R blockade. Thus, both exogenous and endogenous AngII can induce renal cortical hypoxia.

It has been proposed that renal tissue hypoxia is a final common pathway in the pathogenesis of chronic kidney disease (Fine *et al*. 2000; Nangaku, 2006; Tanaka *et al*. 2006; Heyman *et al*. 2008; Palm & Nordquist, 2011). To critically test this hypothesis, we require methods to continuously monitor renal tissue oxygenation in unrestrained animals, so we can elucidate the temporal relationships between renal pathology and renal tissue hypoxia in models of chronic kidney disease such as the Cyp1a1Ren2 rat. Our current findings indicate that telemetric methods are appropriate and valid for this purpose.

## Additional information

### Competing interests

No conflicts of interest, financial or otherwise, are declared by the authors.

### Author contributions

Author contributions: T.W.E., S.C.M., J.A.J., B.J.J., R.G.E. and M.P.K. contributed to the conception and design of research; T.W.E., M.I.P., B.J.J. and M.P.K. performed experiments; T.W.E., M.I.P., C.P.C.O., B.J.J. and M.P.K. analysed data; T.W.E., R.G.E., J.A.J., S.C.M., B.J.J., C.T.P.K. and M.P.K. interpreted results of experiments; T.W.E. and M.P.K. prepared figures; T.W.E. and M.P.K. drafted manuscript; T.W.E., R.G.E., J.A.J., S.C.M., B.J.J., C.T.P.K. and M.P.K. edited and revised manuscript; T.W.E., M.I.P., C.P.C.O., R.G.E., J.A.J., S.C.M., B.J.J., C.T.P.K. and M.P.K. approved final version of manuscript.

### Funding

This work was supported by the European Union, Seventh Framework Programme, Marie Curie Actions (ReTeBESKO ‐ No. 282821 and CARPEDIEM ‐ No 612280), the British Heart Foundation (No. FS/14/2/30630), the Dutch Kidney Foundation (Grant KJPB12.29 and KSBP 10.016) a ZonMwClinical Fellowship (No. 40007039712461), the National Health and Medical Research Council of Australia (1024575) and the University of Auckland (No.9396873).
